# Centering Indigenous Knowledges and Worldviews: Applying the Indigenist Ecological Systems Model to Youth Mental Health and Wellness Research and Programs

**DOI:** 10.3390/ijerph19106271

**Published:** 2022-05-21

**Authors:** Victoria M. O’Keefe, Jillian Fish, Tara L. Maudrie, Amanda M. Hunter, Hariata G. Tai Rakena, Jessica Saniġaq Ullrich, Carrie Clifford, Allison Crawford, Teresa Brockie, Melissa Walls, Emily E. Haroz, Mary Cwik, Nancy Rumbaugh Whitesell, Allison Barlow

**Affiliations:** 1 Johns Hopkins Center for American Indian Health, Department of International Health, Bloomberg School of Public Health, Johns Hopkins University, Baltimore, MD 21231, USA; tmaudri1@jh.edu (T.L.M.); htairak1@jh.edu (H.G.T.R.); mwalls3@jh.edu (M.W.); eharoz1@jh.edu (E.E.H.); mcwik1@jh.edu (M.C.); abarlow@jhu.edu (A.B.); 2 Center for Care Delivery and Outcomes Research, Minneapolis Veterans Affairs Health Care System, Minneapolis, MN 55417, USA; jillian.fish@va.gov; 3 Center for Health Equity Research, Northern Arizona University, Flagstaff, AZ 86011, USA; amanda.urbina@nau.edu; 4 School of Social Work, University of Alaska Anchorage, Anchorage, AK 99508, USA; jsullrich@alaska.edu; 5 Department of Psychology, University of Otago, Dunedin 9016, New Zealand; clica904@student.otago.ac.nz; 6 Department of Psychiatry, University of Toronto, Toronto, ON M5G 1X5, Canada; allison.crawford@utoronto.ca; 7 School of Nursing, Johns Hopkins University, Baltimore, MD 21231, USA; tbrocki1@jhu.edu; 8 Centers for American Indian and Alaska Native Health, Colorado School of Public Health, University of Colorado Anschutz Medical Campus, Aurora, CO 80045, USA; nancy.whitesell@cuanschutz.edu

**Keywords:** Indigenous peoples, well-being, culture, mental health, Indigenous health

## Abstract

Globally, Indigenous communities, leaders, mental health providers, and scholars have called for strengths-based approaches to mental health that align with Indigenous and holistic concepts of health and wellness. We applied the Indigenist Ecological Systems Model to strengths-based case examples of Indigenous youth mental health and wellness work occurring in CANZUS (Canada, Australia, New Zealand, and United States). The case examples include research, community-led programs, and national advocacy. Indigenous youth development and well-being occur through strengths-based relationships across interconnected environmental levels. This approach promotes Indigenous youth and communities considering complete ecologies of Indigenous youth to foster their whole health, including mental health. Future research and programming will benefit from understanding and identifying common, strengths-based solutions beyond narrow intervention targets. This approach not only promotes Indigenous youth health and mental health, but ripples out across the entire ecosystem to promote community well-being.

## 1. Introduction

### 1.1. Mental Health as an Essential Component of Health and Well-Being

Mental health is a vital and inseparable component of health and well-being for Indigenous communities throughout the lands now-called Canada, Australia, New Zealand, and the United States (CANZUS). Te Whare Tapa Whā [1], an influential Māori mental health model from New Zealand, acknowledges four cornerstones of Māori health, Taha tinana (physical health), Taha wairua (spiritual health), Taha whānau (family health and social relationships), and Taha hinengaro (mental health), the foundation of which is connection to whenua (land). The First Nations Mental Wellness Continuum Framework developed in Canada includes a model that centers the interconnectedness of mental health, physical health, emotional health, and spiritual health and well-being [2]. Other American Indian/Alaska Native health and wellness models similarly demonstrate this longstanding knowledge that mental health is facilitated by the balance between mind, body, spirit, and context (e.g., family, community, and culture) [3]. In Australia, the National Strategic Framework for Aboriginal and Torres Strait Islander Peoples’ Mental Health and Social and Emotional Well-Being includes guiding principles underscoring the “whole-of-life view of health” recognized by Aboriginal and Torres Strait Islander communities [4]. This framework outlines four interrelated areas, including mental, physical, spiritual, and cultural health, and further asserts that land is fundamental to wellness [4].

While these views of health and well-being derive from vastly diverse Indigenous peoples from communities and cultures across the world, there is congruence that mental health is intrinsic and integrated with health and wellness. Indigenous mental health and well-being also includes connectedness with ancestors, family, community, and lands, as well as storytelling, ceremony, spirituality, cultural identity and engagement, and self-determination [5,6,7,8,9]. There is a shared understanding that when domains of health and wellness, including mental health, are cultivated and cultural knowledges and practices are followed, Indigenous individuals, families, and communities can strive to be and live well [5,10]. In this paper, we describe strengths-based case examples of Indigenous youth mental health and wellness research, community-led programs, and national advocacy and apply them using a novel Indigenist social-ecological model. This model aligns with Indigenous worldviews of health and wellness in Canada, Australia, New Zealand, and the United States, and can guide future mental health research and programming to understand Indigenous youth well-being across interconnected environmental levels.

### 1.2. Strengths-Based Approaches to Mental Health and Wellness

Indigenous communities, leaders, mental health providers and clinicians, and scholars have called for strengths-based approaches to mental health and wellness that align with traditional views of healing and wellness [6]. Such approaches oppose deficit, risk-focused, individualized, and pathologizing narratives that have been over-used in Indigenous-health research and intervention development with Indigenous peoples [11,12]. Deficit-based approaches also threaten to perpetuate colonialism, both by assuming that Indigenous communities are inferior and by proposing that the solution is to import Western approaches to correct these failings, thus further suppressing Indigenous knowledges and practices [11,13].

Strengths-based approaches inherently contextualize Indigenous mental health and wellness through understanding of historical, intergenerational, social, cultural, and political contexts that support existing community strengths, uphold self-determination and sovereignty, and promote justice and equity [6,11,14,15,16]. A conceptual approach that is more synonymous with and inclusive of Indigenous worldviews on well-being are social-ecological frameworks. Social-ecological frameworks are deeply contextual as they reflect that individuals are embedded within various environmental domains, including family, community, and broader societal and political contexts [6,11,14,17,18].

### 1.3. Indigenizing Ecological Models

**Figure 1:**
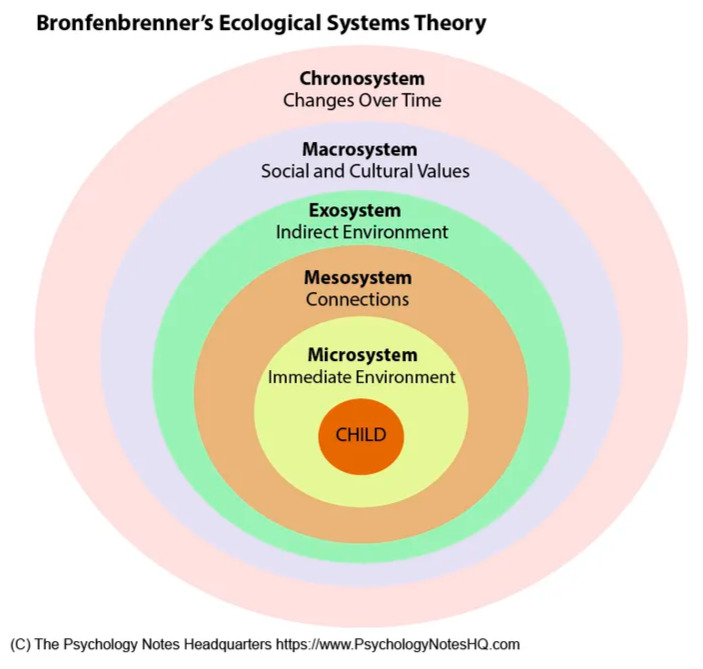
Bronfenbrenner’s Ecological Systems Model for Youth Development. Adapted with permission from Psychology Notes Headquarters [22].

**Figure 2:**
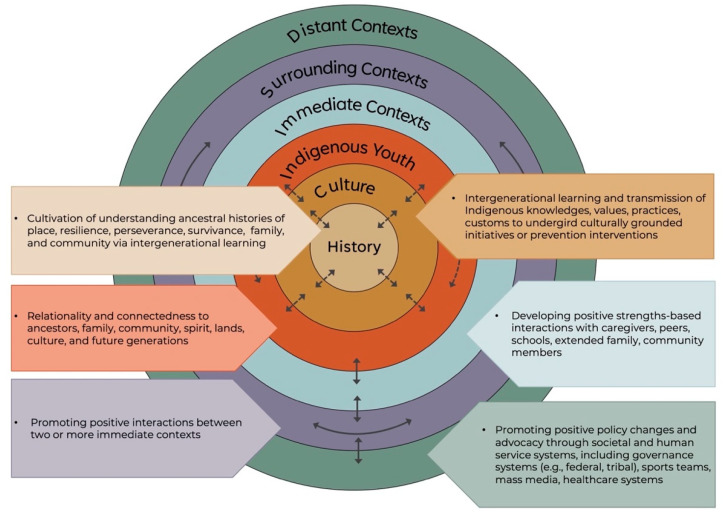
Indigenist Ecological Systems Model. Adapted with permission from [14,17].

Bronfenbrenner’s ecological systems model [19,20] is among the most influential and globally accepted of the social-ecological frameworks [21]. Its intention is to illustrate that youth develop as the result of their interactions with their environments (or ecological systems). In Bronfenbrenner’s model, the individual is situated at the center of a series of nested environments, from the individual at the center, to concentric circles of more distal influences, called the microsystem, mesosystem, exosystem, macrosystem, and chronosystem (See Figure 1) [20]. To make Bronfenbrenner’s model [20] more relevant for Indigenous populations, Fish and Syed [17] developed the Indigenist Ecological Systems Model (see Figure 2) [14], which reconceptualizes the order and meaning of the environments to better account for the influence of historical and cultural contexts on Indigenous peoples’ developmental beginnings and outcomes. By foregrounding Indigenous development in the histories and cultures of Indigenous peoples, the Indigenist model originates in a strengths-based vision of development through interconnectedness and relationality. The Indigenist model was initially used as a theoretical framework to better understand the historical and cultural factors that influence American Indian students’ experiences in higher education [17]. Since then, it has been used as a framework for developing a historically and culturally congruent mental health intervention for Indigenous students [14]. As the findings of these undertakings suggest, Indigenous populations experience the most positive developmental outcomes when they are able to access their histories and cultures in their environments, and less desirable outcomes when their environments prohibit this.

The purpose of this paper is to apply the Indigenist Ecological Systems Model [14] to strengths-based approaches to Indigenous mental health and wellness work occurring in the countries now known as Canada, Australia, New Zealand, and the United States.

## 2. Materials and Methods

Our application of the Indigenist Ecological Systems Model [14] expands upon previous applications to American Indians/Alaska Natives in the United States to Indigenous youth and communities in Canada, Aotearoa/New Zealand, and Australia. We focus on CANZUS (Canada, Australia, New Zealand, United States) because of: shared values of intergenerational, family, community, environmental, and spiritual connectedness, as well as ongoing movements to pass on and/or revitalize Indigenous languages, cultures, and traditions to promote youth and community well-being [8]; similar Indigenous legal and political agreements/treaties with the national governments and self-governance among Indigenous groups [23]; shared similarities in histories of British invasion and settler colonialism resulting in countries today with English being the dominant national language [23]; and similarities in disproportionate mental health inequities (e.g., suicide, psychological distress, depression, and anxiety) endured by Indigenous youth across these four countries compared to non-Indigenous youth, linked to social determinants of health, including colonialism [24,25,26,27]. Though there are similarities and shared experiences, we also recognize the diversity of specific histories, contemporary political and social contexts, and tribal/community cultures both within and between Indigenous communities across these settler colonial countries and that may contribute to differences in health and wellness outcomes.

Below, we describe each level of the Indigenist Ecological Systems Model and apply this model to case examples of strengths-based Indigenous mental health and wellness, including cases from research, community-led programs, and national advocacy across CANZUS (see Figure 2). We purposively highlight case examples outside of Western-based scientific research to honor Indigenous epistemologies, practices, and community-defined evidence as valid and necessary [28]. The authorship team included nine Indigenous and five allied co-authors with vast knowledge of research, community-based programs, and national initiatives. Our approach to purposively selecting case examples was an iterative and collaborative process among all the co-authors. The first author met with the co-authors to discuss each level of the Indigenist Ecological Systems Model to generate initial ideas for case examples. Six of the co-authors searched peer-reviewed literature using PubMed and/or PsycINFO to identify the case examples relevant to each level of the model. To augment the academic literature search, gray literature was also searched using Google, and known Indigenous websites and organizations. Examples were then discussed as part of the paper drafting process. If a case example did not fit, the authors searched for alternatives that fit the model more accurately.

Results from the case examples are discussed below, framing each case’s approaches and findings within each dimension of the Indigenist Ecological Systems Model. Descriptions of the model and case examples are outlined by level in Table 1.

## 3. Application of Case Examples to the Indigenist Ecological Systems Model

### 3.1. Historical Contexts

Historical contexts (i.e., the chronosystem) are the core and third dimension of the Indigenist model [14,38]. Privileging historical contexts in development indicates that histories affect Indigenous peoples’ health and well-being in the past, present, and future. This includes histories of colonialism, histories of resilience and perseverance, and familial, ancestral, and place-based histories that center the importance of lands. The important connection between past, present, and future is a shared teaching among many Indigenous communities in CANZUS [7,8,9,16] and has been used to structure mental health and wellness promotion among youth. Intergenerational engagement encourages Indigenous communities to repair, build, and strengthen relationships between Elders or traditional knowledge holders and youth. Connections between Elders and traditional knowledge holders with youth foster transfer of cultural values, historical cultural knowledge, and observational learning of cultural activities and lifeways. Intergenerational learning and engagement are recognized as imperative to restoring overall health and well-being for Indigenous youth and communities, including mental health [39].

Intergenerational learning is fostered through community, referred to by Cajete as “the living place” [29]. The living place includes immediate family, extended family, clan relatives, the larger tribal community, and lands and the natural environment [29]. These sources of learning are varied and reflect the communal responsibility to ensure Indigenous lifeways persist throughout time. Intergenerational learning occurs through a process that is holistic and integrated with daily living and land-based teachings, differing from the colonial idea of education that often separates learning from other aspects of life [29]. The process of intergenerational learning begins with the development of social structures and relationships and is followed by using creative exploration to foster skills in listening and observation [29]. Storytelling, public speaking, and singing are often used to promote intergenerational learning, while teaching of sacred cultural knowledges may be reserved for ceremonies [29]. Cultural continuity depends on intergenerational learning, which is a lifelong process for Indigenous communities.

As emphasized above, time is crucial to Indigenous flourishing, as connections between the past, present, and future offer access to time-honored knowledges and traditions. This also offers a powerful restorative to historical trauma and losses and protection against ongoing effects of settler colonialism on Indigenous communities and youth [40]. Our examples foreground Elders and knowledge keepers who can provide these connections through a time-honored tradition, the intergenerational transmission of knowledge and oracy [41], exposing Indigenous youth to ancestral and place-based histories to guide them now and for years to come. In addition to important histories, intergenerational learning offers an insight into Indigenous cultural factors—which we discuss next.

### 3.2. Cultural Contexts

Following historical contexts that focus on sources and processes of intergenerational learning, cultural contexts (i.e., the macrosystem) are the second level of the Indigenist model that combine intergenerational learning with cultural practices. Cultural contexts include patterns in beliefs, practices, norms, and customs unique to Indigenous peoples (e.g., language, spirituality) and that give structure to their environments. Cultural contexts are an outgrowth of historical contexts, as various histories (e.g., settler colonial histories and Indigenous histories) mold and shape Indigenous peoples’ cultures. Despite legacies of settler colonialism and violence, Indigenous peoples have maintained or are actively revitalizing their connections to Indigenous knowledges and practices, including connections to lands and cultures [42]. According to the First Nations Mental Wellness Continuum Framework developed in Canada, Indigenous leaders, Elders, youth, and community members affirm that culture is central to mental health wellness [2]. Thus, similar to historical contexts, cultural contexts are relevant to all the environments in the Indigenist model.

When intergenerational engagement and learning (i.e., historical contexts) and cultural activities (i.e., cultural contexts) are combined, prevention against negative mental health inequities occurs. Culturally grounded initiatives to foster intergenerational engagement and cultural knowledge transmission have always existed in Indigenous communities, yet there is growing interest in understanding how these programs promote youth mental health and applying these modalities to prevent mental health inequities (e.g., suicide) through research [43]. One intervention that leverages intergenerational learning and cultural knowledge transfer includes Camp Pigaaq, a camp for Alaska Native youth that provides space for Elders and other guest presenters to share cultural knowledge and teach traditional skills and wellness practices [30]. Participation in Camp Pigaaq has shown to significantly increase positive mood, feelings of belongingness, and perceived coping among Alaska Native youth [30]. Culturally grounded mental health promotion underscores that cultural values and lifeways can be taught through intergenerational engagement to form healthy communities that persist through time.

Language is an important vehicle for passing down culture from generation to generation [44]. Within Aotearoa/New Zealand, Te Kōhanga Reo (Māori immersion language preschools), meaning “the language nest”, is a national movement providing a “culturally structured environment” for child development and aims to strengthen Māori language and culture among youth and future generations [31,44]. During their attendance, Māori children from birth to the of age six are culturally immersed and learn about values, traditions, and language in a warm environment with whānau (extended family). Established in 1982, more than 50,000 children have participated in a Kōhanga Reo, which has been vital to the revitalization of Te Reo Māori (Māori language) and ensuring tamariki (children) grow up and develop immersed in their language and culture [31]. Similar language programs are growing across Indigenous communities. In 2018, the Menominee Indian Tribe of Wisconsin established a language nest named Kaehkēnawapatāēq (Menominee Language Revitalization Program) to train early childhood language teachers [45,46]. Kaehkēnawapatāēq translates to “we learn by observing”, referring to the process of teaching the language to adults while children in the daycare observed and were also immersed in the language nest. The Menominee Indian Tribe of Wisconsin currently has resources and materials for learning history and language that can be accessed in-person or online [32]. Indigenous languages facilitate connectedness to family, community, lands, spirituality, and intergenerational connectedness [8]. In this way, language revitalization is critical to promoting positive mental health, community healing, and wellness [47].

Indeed, historical and cultural contexts are intimately bound with one another. As we have illustrated via case examples, historical contexts provide a foundation through which Indigenous youth gain meaningful access to cultural contexts, including language, spirituality, local values, and various cultural practices and teachings. Historical and cultural contexts converge to provide Indigenous youth with the necessary foundation for living full and healthy lives.

### 3.3. Indigenous Youth

Instead of being at the center of development, Indigenous youth (i.e., the individual level in Bronfenbrenner’s model [20]) are the third level of the Indigenist model [14], including core psychological phenomena (e.g., identity, self-understanding, and self-efficacy). This position indicates that Indigenous histories and cultures come before and are integral to Indigenous youth development. It also signifies that developmental outcomes—both positive and negative—are born from Indigenous youths’ connections to their communities’ histories and evolving cultures.

Within many Indigenous worldviews and cultures, it is impossible to consider an individual separate from their connectedness with other people, lands, and all living beings. Connectedness has been defined as the interrelated welfare of an individual, family, community, and the Earth [48]. To learn more about connectedness and the relational processes that promote child well-being, research was conducted through a literature review of Indigenous communities in Canada, Australia, New Zealand, and the United States [8], and an interview process with 25 Alaska Native knowledge bearers [49]. This research led to the development of an Indigenous Connectedness Framework that describes child well-being as depending on the existence of internal, spirit/culture, family, community, environment, and intergenerational connectedness. These relationships help a child know who they are and where they come from as a relational human being that is interconnected with a collective [8]. When children are perceived as unique beings that are part of a collective, it expands our awareness of well-being to include the wellness of everyone and everything to which they are connected. In this light, when we serve individual children, we are also serving their family, community, the environment, culture/spirit, and ancestors and future generations because who they are is embedded in those interconnected relationships.

It is evident from the Indigenous Connectedness Framework [8] that, as a case example, Indigenous youth experience clear benefits as a result of being immersed in and learning about longstanding cultural practices and traditions. No doubt, these benefits have a ripple effect, extending out to Indigenous families, communities, and transcending the physical universe to an ancestral and spiritual one. Now we turn our attention to the environments that make such historical and cultural connections possible, starting with immediate environments.

### 3.4. Immediate Contexts

Immediate contexts (i.e., the microsystem) are the first level of Bronfenbrenner’s model [20] and the fourth environment in the Indigenist model. Immediate contexts refer to the environments that Indigenous youth have direct interactions with on a regular, ongoing basis [14]. While this can include parents or caregivers, peers, schools, and community (i.e., reservations and urban neighborhoods), it can also refer to extended family depending on the nature of the interactions Indigenous youth have with them [50].

The Thiwáhe Gluwáš’akapi Program (translated as sacred home in which family is made strong) provides a strong example of centering connectedness to and engagement with various levels of environment in promoting mental health and wellness among Indigenous youth. Thiwáhe Gluwáš’akapi was derived from a community-based participatory research substance use prevention study; an Indigenous researcher living and working in the community led the cultural adaptation of the program and paid strong attention to the immediate context of adolescents in the process. The resulting intervention is deeply rooted in family and kinship ties and integrates kinship teachings while emphasizing the relationships, responsibilities, and roles youth hold within their families and their larger communities [33]. Adolescents were enrolled in the study with one caregiver to participate in seven weekly group sessions held at their local school. Kinship ties were emphasized by including extended family beyond the enrolled caregiver in group sessions. Thiwáhe Gluwáš’akapi also promoted tribal values through curricula designed to develop listening skills that align with cultural traditions of oral storytelling and learning [51]. Further, the Thiwáhe Gluwáš’akapi program utilized traditional language for kinship relationship terms to emphasize the interconnectedness of kinship and culture.

What is remarkable about interventions such as these is that they leverage the existing strengths of Indigenous youths’ immediate contexts (i.e., family and community) and build on them through culturally relevant programming. On their own, immediate contexts can have a robust impact on Indigenous youth. However, as we describe next, immediate contexts can also create partnerships with each other to further their impact.

### 3.5. Surrounding Contexts

Surrounding contexts (i.e., the mesosystem) are the second level of Bronfenbrenner’s original model [20], and the fifth level of the Indigenist model [14]. Surrounding contexts are interactions between two or more immediate contexts (e.g., peers and parents) that affect Indigenous youth. Previous research with the Indigenist model indicated that surrounding contexts in the form of partnerships can address structural inequities in Indigenous peoples’ environments, critical to promoting Indigenous youths’ mental health and well-being.

Listening to One Another to Grow Strong (LTOA) is an example of a community-driven and culturally adapted program rooted in the philosophy that family well-being (e.g., microsystem) is foundational for individual and community (microsystem) health [52]. This program was developed through a collaboration between First Nations communities in British Columbia, Manitoba, Ontario, Quebec, and university teams in the United States and Canada. LTOA is designed to be inclusive of the family unit, and therefore the immediate contexts of youth, by providing activities to be completed by a family, as well as youth- and caregiver-specific activities. The family program component of LTOA is delivered across 14 two-and-a-half-hour sessions, while the school program is delivered through 6 one-hour sessions [34,53]. The final lesson of the school program includes feasting with families in schools to highlight the achievements of students and to connect families to youth in their school environments [34]. All sessions are facilitated by a local facilitator and usually in partnership with local Elders, who are provided with an Elder manual designed to orient them to the curriculum and their role in delivery [54]. Qualitative evaluation of the LTOA program found positive impacts on family bonding and communication skills, while quantitative evaluations found positive impacts on youth well-being in the form of reduced feelings of distress and elevated sense of connection to family and community [6]. Therefore, the LTOA program demonstrates how interactions and partnerships between immediate contexts (families, Elders, schools, peers, and communities) can act in synergy to support positive mental health and wellness for Indigenous youth.

Surrounding environments have the potential to overcome barriers to youth accessing their Indigenous histories and cultures. By establishing partnerships and building relationships with other immediate environments (e.g., Elders, communities, families, and peers), schools can develop local and culturally appropriate mechanisms for making Indigenous histories and cultures accessible in places where it matters most. Other immediate environments that are ripe for these types of partnerships include healthcare centers eager to provide suitable and relevant services to Indigenous youth.

### 3.6. Distant Contexts

The application of Bronfenbrenner’s [20] exosystem in the Indigenist Ecological Systems Model [14] depicts social and political contexts that affect Indigenous peoples and their communities. Distant contexts (i.e., the exosystem) are the sixth and final level of the Indigenist model and represent environments that Indigenous peoples may or may not be actively involved in, but are indirectly impacted by these distant contexts. The case examples presented span some of the following critical domains, including the government (e.g., federal government, self-determination, and tribal sovereignty), sports teams’ names and mascots, Indigenous visibility and representation in mass media, and healthcare systems.

Self-determination, community control, and tribal sovereignty have been identified as vital to health, including mental health promotion and well-being across Indigenous communities in CANZUS [5,24,35]. In Aotearoa/New Zealand, tino rangatiratanga is a Māori concept deeply rooted in Māori worldviews and historical contexts representing the essential domains of self-determination, sovereignty, self-governance, and autonomy vital to health and well-being [13,16,55]. Tino rangatiratanga is described as having a cyclical and interdependent relationship with the well-being of an individual and the collective, including whānau (extended families), hapū (sub-tribes), and iwi (tribes), and if supported and promoted nationally, can benefit health and well-being for all New Zealanders [55]. There are other examples demonstrating the potential power of community autonomy, control, and sovereignty in promoting mental health and wellness among Indigenous youth and communities. For example, Chandler and Lalonde [40] documented among First Nations communities in Canada that cultural continuity was related to reduced youth suicide. Specifically, they identified six variables that comprise cultural continuity: assertion of or political movements toward sovereignty over (a) traditional lands; (b) governance; (c) education; (d) law enforcement and first responders and (e) health services; and (f) formally recognized fora, which promote culturally meaningful values and traditions [40]. Among First Nations communities with a higher amount of cultural continuity factors, they observed lower youth suicide rates compared to communities with fewer factors.

Federal policy can also impact culturally safe mental health programming for Indigenous communities. For example, in Australia, cultural competency has been deemed a professional requirement for the national mental health sector working with Aboriginal and Torres Strait Islander clientele [36]. The iterative nature of cultural competency acquisition warrants emphasis, as it is only through sustained dedication toward providing culturally safe services that transformational practice is possible [24]. Further work can be conducted through national policies across CANZUS to align mental health services with Indigenous epistemological and ontological positions, ensure human rights and decolonizing practices, and offer critical reflection tools to support mental health service providers to incorporate such principles into their work [24].

The visibility of Indigenous peoples within society and accurate portrayals may also promote Indigenous youth mental health and well-being. This follows from the research showing that negative portrayals, such as American Indian/Alaska Native sports mascots, have deleterious impacts on American Indian/Alaska Native youth mental health [56]. Within the United States, an Indigenous non-profit organization, IllumiNative [37], is leading initiatives to increase visibility and accurate narratives and portrayals of American Indians/Alaska Natives in the United States. They published the Reclaiming Native Truth Report [57], which underscores how visibility and representation of American Indians/Alaska Natives can be strengthened across multiple forms of media, including social and news media, the entertainment industry, and education.

For decades, settler governments and structures have made decisions that affect Indigenous peoples with limited and insufficient input from Indigenous peoples and tribal nations themselves. As these case examples indicate, there are shifts in this trend wherein Indigenous peoples are asserting their right to sovereignty and self-determination, advocating for new culturally congruent policies and other structural changes, and challenging settler depictions of Indigenous peoples. Collectively, this work aims to create a better tomorrow for future generations of Indigenous youth to develop and thrive.

## 4. Discussion

We applied a novel framework, the Indigenist Ecological Systems Model [14], to positive case examples of Indigenous youth mental health and wellness research, community-led programs, and national initiatives in Canada, Australia, New Zealand, and the United States that reflect a deep contextual and cultural understanding of Indigenous conceptualizations of mental health and well-being. This framework recognizes that Indigenous youth development and well-being occur through strengths-based relationships across interconnected environmental levels [14]. By utilizing an Indigenous framework and strengths-based case examples, we resisted deficit and pathologizing narratives that tend to dominate health research with or about Indigenous peoples [11,13]. While social-ecological frameworks have been critiqued due to positioning health as a goal [11], our approach aimed to describe broad and multi-level initiatives that naturally promote Indigenous youth mental health and well-being (e.g., visibility and positive representation; self-determination). Further, this approach respected the interconnected nature of physical, mental, emotional, and spiritual health, and connection to family, community, and larger contexts that are common among Indigenous communities in Canada, Australia, New Zealand, and the United States [5].

We purposely included positive examples of Indigenous-led, community-based programs, and national initiatives outside of Western research to take “a comprehensive view of what constitutes evidence beyond colonial constructs” [58]. While many of these programs are familiar at community levels, they may be unfamiliar or missing from larger ecosystems of health research. For example, within distant contexts, we highlighted the work of IllumiNative, an Indigenous-led non-profit organization in the United States that seeks to increase visibility and positive representations of American Indians/Alaska Natives throughout society [37]. There is empirical research linking negative psychosocial impacts experienced by American Indian/Alaska Native youth and adults to negative stereotypes about American Indians/Alaska Natives [56]. However, understanding how societal visibility and positive representations promote American Indian/Alaska Native health, mental health, and holistic well-being has been largely absent from Indigenous health research. Research and advocacy can promote Indigenous interests, positive outcomes, and social and political change [13]—in this case, through promoting Indigenous youth mental health and overall well-being, which has an undeniable connection to promoting Indigenous communities’ wellness [8].

### 4.1. Indigenous Ecologies of Health and Wellness

Our application of the Indigenist model revealed several notable findings. An essential theme that cuts across all case examples is: Indigenous peoples are taking into consideration the complete ecologies of Indigenous youth to foster their holistic health [3]. Rather than simply considering the health of the individual, we see Indigenous peoples creating innovative approaches to gifting Indigenous youth with the intergenerational and cultural foundation that is necessary for living full and meaningful lives [5]. These approaches harness Indigenous histories and cultures across places and spaces that are crucial to Indigenous youth development—immediate environments, such as family, school, Elders, and community; surrounding environments, such as school-Elder partnerships; and distant environments, such as tribal self-governance and policy. Taken together, Indigenous approaches to whole health weave together the various environments in the Indigenist model, creating a generative network of health that encompasses Indigenous families and communities across past, present, and future generations [11].

Further, we used a cross-Indigenous approach, rather than a cross-cultural approach that is often applied in psychology and mental health fields [59]. Indeed, “the spiritual, creative, and political resources that Indigenous peoples can draw on from each other provide alternatives for each other” [13]. Toward that end, we provided distinct case examples from different countries and tribal communities, yet drew similarities that demonstrate an engagement across levels of the Indigenist Ecological Systems Model [14]. At times, it was challenging to determine which environment a particular program belonged to, given the degree of overlap between the levels. However, this lends greater support to Indigenous concepts of health and wellness as holistic and comprehensive, spanning multiple environments. Ultimately, these health-related programs shine a light on how Indigenous peoples are cultivating cultures of health rooted in their knowledges [10] that can inform future research and interventions.

### 4.2. Future Directions

Fostering Indigenous youth well-being is integral to the future of Indigenous communities [8]. While each section of the Indigenist model holds promise for Indigenous health, its power lies in what it collectively offers across environments. The Indigenist model further elucidates what Indigenous peoples have been voicing and advocating for since time immemorial—that there are countless strengths within Indigenous communities that, when channeled, enable Indigenous youth to thrive [6,15,16]. It is now time for federal governments, policymakers, funders, and health researchers to support initiatives that embolden Indigenous lifeways as legitimate strengths and health approaches, albeit long overdue.

There are various Indigenous-led solutions to increasing and enhancing the strengths of entire Indigenous communities and fostering inherent strengths of Indigeneity. This includes creating local and context-specific curricula for teaching language and cultural beliefs and practices, creating opportunities to connect youth with Elders and other knowledge keepers, developing or adapting culture-forward interventions, and engaging in national efforts that draw on Indigenous ecologies to make sociopolitical changes across the public landscape. Although some interventions (e.g., Thiwáhe Gluwáš’akapi [33]) were developed to address one priority area (i.e., substance use prevention) among Indigenous youth, such programs often can be applied to address the root causes of other concerns (i.e., suicide prevention). However, note that the shared paths are rooted in Indigenous strengths and how to bolster them, rather than targeting reductions in “problematic” outcomes. Accordingly, future research will benefit from understanding the ways in which Indigenous-led programs promote the collective health and well-being of youth beyond narrow targets of intervention. Future research must focus on identifying common, strengths-based solutions for promoting mental health to not only promote well-being for Indigenous youth, but ripple out across their entire ecosystem.

## 5. Conclusions

The case examples we selected from Indigenous research and community-based programs illustrate the Indigenist Ecological Systems Model and demonstrate the alignment of the model with Indigenous concepts of health and wellness. Through this social-ecological model, mental well-being is understood from a vantage of holism, where the individual is understood within a highly relational context, interconnected with historical and cultural contexts, and with ancestors, family, community, spirit, lands, and future generations. The flexibility of the Indigenist Ecological Systems Model demonstrates its utility to guide the development of Indigenous youth health and mental health research, interventions, and programs. Using this model centers Indigenous knowledges and worldviews, and will help to ensure that these perspectives guide the future research and program development aimed at youth mental health and well-being, with attention to each social-ecological level and the interconnections between them. Although we highlighted projects from across global Indigenous contexts, it remains critical for such research to be Indigenous-led and grounded in the specificity of local place and culture. By focusing on complete ecologies and domains of strength, Indigenous-led research and action is leading the way in advancing Indigenous youth well-being and promoting flourishing within and by communities.

## Figures and Tables

**Table 1:** Indigenist Ecological Systems Model description of levels and applied case examples.

Level of Indigenist Ecological Systems Model	Definition of How Contexts Operate	Select Examples	Location of Examples
Historical Contexts	Cultivation of understanding of ancestral histories of place, resilience, perseverance, family, and community development via intergenerational learning. Additionally, the importance of understanding survivance, perseverance, and healing in context of historical trauma and loss, and colonialism	Intergenerational connection between past, present, and future [8]	Canada, Australia, New Zealand, United States [8]
		Storytelling as a method of intergenerational learning and knowledge transmission [29]	United States [29]
Cultural Contexts	Intergenerational learning and transmission of Indigenous cultural knowledges, values, practices, customs to undergird culturally grounded initiatives and/or prevention interventions	Camp Pigaaq: Elders share cultural knowledge and traditions with youth [30]	United States [30]
		Te Kōhanga Reo: Māori immersion language preschools [31]	New Zealand [31]
		Kaehkēnawapatāēq: language revitalization program, Menominee Indian Tribe of Wisconsin [32]	United States [32]
Individual	Understanding that individuals are relational beings and interconnected with ancestors, family, community, environment, spirit, past, present, and future generations	Youth well-being is dependent upon internal, spiritual, cultural, family, community, environmental, historical, and intergenerational connectedness [8]	Canada, Australia, New Zealand, United States [8]
Immediate Contexts	Developing positive strengths-based interactions with caregivers, peers, schools, extended family, and community members	Thiwáhe Gluwáš’akapi: an adolescent community-engaged substance use prevention intervention grounded in family and kinship teachings to emphasize family and community relationships, responsibilities, and roles [33]	United States [33]
Surrounding Contexts	Promoting positive interactions between two or more immediate contexts	Listening to One Another Grow Strong: a culturally adapted program that includes activities that span across youth, caregiver, school, and Elder communities [34]	Canada [34]
Distant Contexts	Promoting positive policy changes and advocacy through societal and human service systems, including governance systems (e.g., federal and tribal), sports teams, mass media, and healthcare systems	Self-determination, community control, tribal sovereignty [5,24,35]	Canada, Australia, New Zealand, United States [5,24,35]
		Federal policy to enact culturally safe healthcare programming for Indigenous communities [24,36]	Australia [24,36]
		Advocacy to increase visibility and accurate representations of Indigenous peoples across sectors of national society, including media [37]	United States [37]

## Data Availability

Not applicable.
